# EMT-Regulome: a database for EMT-related regulatory interactions, motifs and network

**DOI:** 10.1038/cddis.2017.267

**Published:** 2017-06-15

**Authors:** Zhangxiang Zhao, Wenbin Zhou, Yue Han, Fuduan Peng, Ruiping Wang, Ruihan Yu, Chengyu Wang, Haihai Liang, Zheng Guo, Yunyan Gu

**Affiliations:** 1 College of Bioinformatics Science and Technology, Department of Systems Biology, Harbin Medical University, Harbin 150086, China; 2 Training Center for Students Innovation and Entrepreneurship Education, Harbin Medical University, Harbin 150086, China; 3 Department of Pharmacology, Harbin Medical University, Harbin 150086, China; 4 Key Laboratory of Ministry of Education for Gastrointestinal Cancer, Department of Bioinformatics, Fujian Medical University, Fuzhou 350004, China; 5 Fujian Key Laboratory of Tumor Microbiology, Fujian Medical University, Fuzhou 350004, China

*Dear Editor*,

The Epithelial-mesenchymal transition (EMT) is a fundamental cellular process that governs biological processes, such as embryonic development, tumor cell metastasis, organ fibrosis and tissue regeneration.^1^ EMT allows polarized, immotile epithelial cells to transform into motile mesenchymal cells.^2^ This switch is mediated by regulatory factors such as transcription factor (TF), microRNA (miRNA) and long non-coding RNA (lncRNA) at transcriptional and post-transcriptional levels.^3, 4^ These regulatory factors play co-regulational roles on the EMT process and the regulatory loops act as motifs, which are the basic units in the regulatory network.^5^ However, a database with comprehensive EMT regulatory relationships is still lacking. EMT-Regulome comprehensively collected the interactions between regulators and targets, including TF-target, miRNA-target, miRNA-lncRNA, TF-lncRNA and TF-miRNA and identified 10 types of co-regulation motifs for EMT process (Figure 1). EMT-Regulome provides the option for searching EMT-related regulatory interactions, motifs and networks, which facilitates the investigations of the mechanisms of EMT involved in diseases, such as tumor cell invasion. The database is freely available at http://www.medsysbio.org/EMTRegulome.

In current version, EMT-Regulome contains 1740 miRNAs, 8783 lncRNAs and 13 025 coding genes, which includes 370 TF coding genes and 350 EMT-related genes (Supplementary Figure 1A). We collected regulatory relationships, including TF-targets, miRNA-targets, miRNA-lncRNA, TF-lncRNA and TF-miRNA, from public databases (Figure 1). Then, we integrated the regulation into a comprehensive network and searched 10 types of motifs that are the basic units in the regulatory network (Figure 1). Especially, for the fifth motif type, known as competing endogenous RNA (ceRNA), we used hypergeometric test to measure whether two RNA components significantly shared miRNAs. The motifs containing at least one EMT-related gene from dbEMT,^6^ which collected experimentally verified EMT-related genes from literatures, were defined as EMT-related motifs in the EMT-Regulome database. The statistics of motifs are shown in Figure 1. The EMT-Regulome database provides a user-friendly interface that allows users to conveniently search, browse and download data (Supplementary Figure 2). In the ‘Search’ page, the query function can be performed using Symbol or ID of coding gene/miRNA/lncRNA with specified motif types. The search results are composed of three parts:(i) information box of gene, miRNA or lncRNA, which is offered as a search condition, including brief functional description, location in chromosome and external link to NCBI, miRBase or Ensembl for more detail; (ii) motifs are grouped by types, and source databases of their regulation are provided. (iii) returned motifs could be visualized as a network and are allowed to be exported as PNG files in various layouts by the Cytoscape web tool (http://js.cytoscape.org).

As a case analysis, the EMT-Regulome database was applied to identify potential activated EMT motifs in the mesenchymal subtype of ovarian cancer by employing expression profiles from The Cancer Genome Atlas (TCGA). Yang *et al.*^7^ have classified the TCGA ovarian cancer samples into integrated epithelial (iE) subtype and integrated mesenchymal (iM) subtype labels. Here, the activated motifs were defined as the nodes in motifs that were differentially expressed in iM compared with the iE ovarian cancer samples (*T*-test) and the edges in motifs were differentially co-expressed in iM type (Pearson correlation test). All the activated EMT-related motifs in ovarian cancer are available in the download page or by searching specific nodes with statistical control in search page.

In conclusion, EMT-Regulome provides a comprehensive landscape of EMT-related regulation motifs to facilitate the investigation of the mechanism of EMT involved in the pathological and physiological processes. The features of EMT-Regulome include, (i) comprehensively integrated different EMT-related regulatory relationships from multiple databases; (ii) systematically provided EMT-related motifs, such as feed-forward loop and ceRNA with statistical control; (iii) all the EMT-related regulatory relationships can be visualized as network and be downloaded.

## Figures and Tables

**Figure 1:**
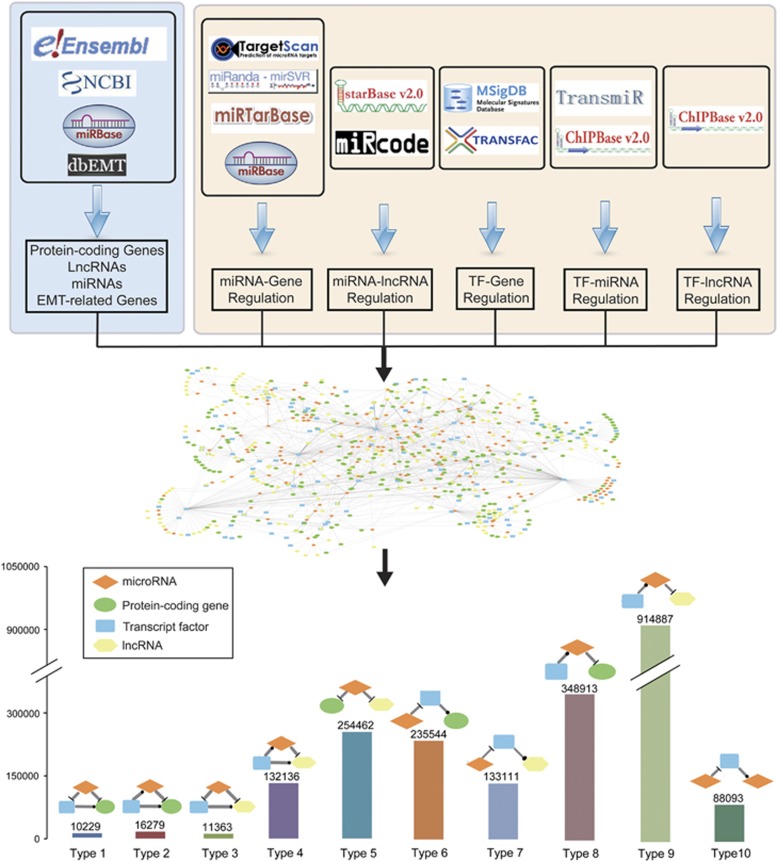
The flow chart of the EMT-Regulome database
