# Supplementary material for: EMT-Regulome: a database for EMT-related regulatory interactions, motifs and network
Source: Cell Death Dis. 2017 Jun 15;8(6):e2872–. doi: 10.1038/cddis.2017.267 (PMC5520922; doi:10.1038/cddis.2017.267)
Supplement: Supplementary Information [file cddis2017267x1.docx]

**Supplementary Information for**

**EMT-Regulome: a database for EMT-related regulatory interactions, motifs and network**

Zhangxiang Zhao^1,2#^, Wenbin Zhou^1#^, Yue Han^1^, Fuduan Peng^1^, Ruiping Wang^1^, Chengyu Wang^1,2^, Ruihan Yu^1^, Haihai Liang^3^, Zheng Guo^1,4,5*^ and Yunyan Gu^1,2*^

^1^College of Bioinformatics Science and Technology, Harbin Medical University, Harbin, 150086, China

^2^Training Center for Students Innovation and Entrepreneurship Education, Harbin Medical University, Harbin, 150086, China

^3^Department of Pharmacology, Harbin Medical University, Harbin, 150086, China

^4^Key Laboratory of Ministry of Education for Gastrointestinal Cancer, Department of Bioinformatics, Fujian Medical University, Fuzhou, 350004, China

^5^Fujian Key Laboratory of Tumor Microbiology, Fujian Medical University, Fuzhou, 350004, China

^#^Contribute equally to this work.

*Corresponding author: Yunyan Gu or Zheng Guo, College of Bioinformatics Science and Technology, Harbin Medical University, Harbin, 150081, China. E-mail: [guyunyan@ems.hrbmu.edu.cn](mailto:guyunyan@ems.hrbmu.edu.cn) or [guoz@ems.hrbmu.edu.cn](mailto:guoz@ems.hrbmu.edu.cn)

**Section I: Data Collection**

Protein-coding genes of Homo Sapiens were obtained from the NCBI database^1^. LncRNAs, including “lincRNA”, “antisense”, “processed transcript”, “sense intronic” and “To be Experimentally Confirmed (TEC)” were acquired from Gencode (Release 24)^2^. Mature miRNAs with ID and their miRNA precursors were downloaded from miRBase (Release 21)^3^. A total of 377 EMT-related genes were obtained from dbEMT(www.dbemt.bioinfo-minzhao.org), which collected experimentally verified genes from literatures^4^. To comprehensively collect regulatory relationships among TFs (Transcription factors), miRNAs (microRNAs), lncRNAs (long non-coding RNAs) and targets, we collected five types of regulation (TF-target, miRNA-target, miRNA-lncRNA, TF-lncRNA and TF-miRNA) from different public databases (Supplementary Figure 1). The miRNA-targets interactions were downloaded from TargetScan (Release 7.0)^5^, miRBase (Release v5)^6^, miRanda (August 2010 release)^7^ and miRTarBase (Release 6.0)^8^.The miRNA-target interactions recorded in the database of miRTarBase were validated by reporter assay, western blot, microarray and next-generation sequencing experiments. For TargetScan, miRBase and miRanda, we selected miRNA-predicted targets regulation based on the evolutionary conservation among mammalians and good score of sequencing match between miRNA and mRNA. Considering that miRNA-targets interactions predicted by multiple algorithms might be more reliable, miRNA-targets interactions appearing in at least two databases were included in our work. We collected TF-target interactions from the MSigDB database with C3 transcription factor targets gene sets based on the the known evolutionarily conserved interactions in mammals (March 2015 release)^9^ and TRANSFAC^10^. We downloaded miRNA-lncRNA interactions from StarBase v2.0 (September 2013 release)^11^ and collected miRNA-lncRNA interaction from miRcode with supports by CLIP-Seq experiments. Then, we obtained TF-lncRNA interactions and TF-miRNA interactions from ChIPBase (November 2012 release)^12^, which identified regulatory relationships based on ChIP-Seq data. Finally, TF-miRNA regulatory relationships, which were supported by experiments, were obtained from TransmiR (January 2013 release)^13^. The detailed statistics of data used in EMT-Regulome database were showed in Supplementary Figure 1.

**Section II: Data Integration**

All the interactions of TF-target, miRNA-target, miRNA-lncRNA, TF-lncRNA and TF-miRNA, which were collected from public databases, are integrated into a regulatory network. Then, we searched for ten types of motifs in the integrated regulatory network, which includes both loop motifs and linear motifs. Here, in this study, we only focused on the motifs with three nodes. The first motif type is miRNA-mediated loop, in which a protein-coding gene is the common target of miRNA and TF. The second motif type is TF-mediated loop, in which a protein-coding gene is the common target of miRNA and TF. Above two motif types, known as feed-forward loops (FFLs), represent a combination of TF and miRNA regulation. FFLs enhance the activation or repression of the common target genes of TFs and miRNAs at both transcriptional and post-transcriptional levels, which improve the stability of the concentrations of target genes^14, 15^. The third motif type is miRNA-mediated loop, in which a lncRNA is the common target of miRNA and TF, while the forth motif type is TF-mediated loop, in which a lncRNA is the common target of miRNA and TF. The reciprocal regulatory circuit among TF, miRNA and lncRNA could coordinately regulate the status of cells, such as promotion of breast cancer stem cell maintenance^16^. The fifth motif type, known as a relationship of competing endogenous RNA (ceRNA), represents that lncRNAs could competitively bind to miRNAs to affect the expression level of protein-coding genes, who shared the binding sites of miRNAs with the lncRNA^17^. And, the ceRNAs have been reported as important roles in the process of EMT^18, 19^. For example, lncRNA H19 functioned as a ceRNA for miR-138 and miR-200a, antagonized functions of miRNAs and led to the de-repression of miRNAs’ endogenous targets ZEB1 and ZEB2, which promoted EMT in colon cancer cells^18^. The hypergeometric test was used to test whether the lncRNAs and protein-coding genes significantly shared miRNAs. The *P* values were adjusted by Benjamini-Hochberg (BH) method and ceRNAs with FDR<0.05 were retained in the database. The sixth to tenth motif types are linear regulation relationship, which are popular regulatory mechanisms in biological processes and diseases^20^. When the TF and the target are the same in the eighth motif type, and the miRNAs in the tenth motif type are the same, the eighth and tenth motif types are the feedback loops (FBLs), which could generate mutually exclusive expression of the miRNA and TF and act as toggle switches between two different cell status^15^.

## Section III: Construction of EMT-Regulome

The popular Linux, Apache, MySQL and Python (LAMP) architecture was implemented to build the dynamic database backend. The EMT-Regulome database is implemented using HTML and Django, a high-level Python Web framework, with MySQL server. The interface components are designed and implemented in Cascading Styling Sheet (CSS) and Javascript, which have been tested in the Google Chrome and Firefox browsers.

All the motifs containing at least one EMT-related gene from dbEMT were defined as EMT-related motifs in the EMT-Regulome database. The EMT-Regulome database provides an user-friendly interface that allows users to conveniently search, browse and download data (Supplementary Figure 2). In the “Search” page, the query function is composed of the basic search, including Symbol or ID of gene/miRNA/lncRNA, and other optional search with specific motif type and statistical control. The search results are composed of three parts: (i) Information box of gene, miRNA or lncRNA, which was set as a keyword, including its brief functional description, location in chromosome and external link to NCBI, miRBase or Ensembl for more detail; (ii) Motifs are grouped by types and source databases of their regulation are indicated. Numbers in tags indicate that the number of records for this motif. All these results are allowed to export as an Excel file; (iii) Returned motifs could be visualized as network graph for each type and the network is allowed to be exported as PNG file in various layouts. Additionally, in the “Browse” page, users can also browse all motifs as sorted by their types. Coding genes, miRNAs and lncRNAs included in the EMT-Regulome are also listed with their corresponding external links to the source databases with concrete information. In the “Download” page, users are permitted to download all EMT-related motifs and activated motifs in mesenchymal ovarian cancer by the form of tab-delimited text file. And, a detailed tutorial showing how to use EMT-Regulome is available on the “About” page.

**Section IV: Application using TCGA data**

As a case analysis, we applied the EMT-Regulome database on the expression profiles of ovarian cancer from The Cancer Genome Atlas (TCGA). The analysis aimed to investigate the deregulation of EMT-motifs in the progress of EMT in ovarian cancers. We downloaded the level 3 of 586 mRNA and 587 miRNA expression profiles of ovarian cancers from TCGA data portal. A total of 412 ovarian cancer samples covering 12717 lncRNAs were collected from TANRIC^21^. Only lncRNAs with non-zero values in more than 90% samples were retained. The final lncRNA expression profile consists of 3574 lncRNAs and 412 samples. Yang *et al.* have classified the TCGA ovarian cancer samples into integrated epithelial (iE) subtype and integrated mesenchymal (iM) subtype labels^22^. Comparing epithelial samples with mesenchymal samples, the t-test was performed to identify differentially expressed genes, miRNAs and lncRNAs, then the *P* values were adjusted by the BH method. The correlations of each edge between genes, miRNAs or lncRNAs in the EMT-related motifs were evaluated by the Pearson Correlation Coefficient in epithelial and mesenchymal samples, respectively. All procedures were implemented in R-3.1.3 (www.r-project.org). The results of differential expression and correlation analyses are available on the “Download” interface in the database of EMT-Regulome.

**Reference**

1. Maglott D, Ostell J, Pruitt KD, Tatusova T. Entrez Gene: gene-centered information at NCBI. *Nucleic acids research* 2011, **39**(Database issue): D52-57.

2. Harrow J, Frankish A, Gonzalez JM, Tapanari E, Diekhans M, Kokocinski F, et al. GENCODE: the reference human genome annotation for The ENCODE Project. *Genome research* 2012, **22**(9): 1760-1774.

3. Griffiths-Jones S. miRBase: the microRNA sequence database. *Methods in molecular biology* 2006, **342**: 129-138.

4. Zhao M, Kong L, Liu Y, Qu H. dbEMT: an epithelial-mesenchymal transition associated gene resource. *Scientific reports* 2015, **5**: 11459.

5. Agarwal V, Bell GW, Nam JW, Bartel DP. Predicting effective microRNA target sites in mammalian mRNAs. *eLife* 2015, **4**.

6. Griffiths-Jones S, Grocock RJ, van Dongen S, Bateman A, Enright AJ. miRBase: microRNA sequences, targets and gene nomenclature. *Nucleic acids research* 2006, **34**(Database issue): D140-144.

7. Betel D, Wilson M, Gabow A, Marks DS, Sander C. The microRNA.org resource: targets and expression. *Nucleic acids research* 2008, **36**(Database issue): D149-153.

8. Hsu SD, Lin FM, Wu WY, Liang C, Huang WC, Chan WL, et al. miRTarBase: a database curates experimentally validated microRNA-target interactions. *Nucleic acids research* 2011, **39**(Database issue): D163-169.

9. Subramanian A, Tamayo P, Mootha VK, Mukherjee S, Ebert BL, Gillette MA, et al. Gene set enrichment analysis: a knowledge-based approach for interpreting genome-wide expression profiles. *Proceedings of the National Academy of Sciences of the United States of America* 2005, **102**(43): 15545-15550.

10. Matys V, Fricke E, Geffers R, Gossling E, Haubrock M, Hehl R, et al. TRANSFAC: transcriptional regulation, from patterns to profiles. *Nucleic acids research* 2003, **31**(1): 374-378.

11. Li JH, Liu S, Zhou H, Qu LH, Yang JH. starBase v2.0: decoding miRNA-ceRNA, miRNA-ncRNA and protein-RNA interaction networks from large-scale CLIP-Seq data. *Nucleic acids research* 2014, **42**(Database issue): D92-97.

12. Yang JH, Li JH, Jiang S, Zhou H, Qu LH. ChIPBase: a database for decoding the transcriptional regulation of long non-coding RNA and microRNA genes from ChIP-Seq data. *Nucleic acids research* 2013, **41**(Database issue): D177-187.

13. Wang J, Lu M, Qiu C, Cui Q. TransmiR: a transcription factor-microRNA regulation database. *Nucleic acids research* 2010, **38**(Database issue): D119-122.

14. Riba A, Bosia C, El Baroudi M, Ollino L, Caselle M. A combination of transcriptional and microRNA regulation improves the stability of the relative concentrations of target genes. *PLoS computational biology* 2014, **10**(2): e1003490.

15. Zhang HM, Kuang S, Xiong X, Gao T, Liu C, Guo AY. Transcription factor and microRNA co-regulatory loops: important regulatory motifs in biological processes and diseases. *Briefings in bioinformatics* 2015, **16**(1): 45-58.

16. Peng F, Li TT, Wang KL, Xiao GQ, Wang JH, Zhao HD, et al. H19/let-7/LIN28 reciprocal negative regulatory circuit promotes breast cancer stem cell maintenance. *Cell death & disease* 2017, **8**(1): e2569.

17. Salmena L, Poliseno L, Tay Y, Kats L, Pandolfi PP. A ceRNA hypothesis: the Rosetta Stone of a hidden RNA language? *Cell* 2011, **146**(3): 353-358.

18. Liang WC, Fu WM, Wong CW, Wang Y, Wang WM, Hu GX, et al. The lncRNA H19 promotes epithelial to mesenchymal transition by functioning as miRNA sponges in colorectal cancer. *Oncotarget* 2015, **6**(26): 22513-22525.

19. Lv J, Fan HX, Zhao XP, Lv P, Fan JY, Zhang Y, et al. Long non-coding RNA Unigene56159 promotes epithelial-mesenchymal transition by acting as a ceRNA of miR-140-5p in hepatocellular carcinoma cells. *Cancer letters* 2016, **382**(2): 166-175.

20. Cao H, Xu E, Liu H, Wan L, Lai M. Epithelial-mesenchymal transition in colorectal cancer metastasis: A system review. *Pathology, research and practice* 2015, **211**(8): 557-569.

21. Li J, Han L, Roebuck P, Diao L, Liu L, Yuan Y, et al. TANRIC: An Interactive Open Platform to Explore the Function of lncRNAs in Cancer. *Cancer research* 2015, **75**(18): 3728-3737.

22. Yang D, Sun Y, Hu L, Zheng H, Ji P, Pecot CV, et al. Integrated analyses identify a master microRNA regulatory network for the mesenchymal subtype in serous ovarian cancer. *Cancer cell* 2013, **23**(2): 186-199.

**Supplementary Figure 1-Statistics of EMT-Regulome.**

**(A)**The number of coding genes, lncRNAs and miRNAs in the database of EMT-Regulome.

**(B)**The number of regulation types in the database of EMT-Regulome.

**(C)**The number of regulation from public databases.

**
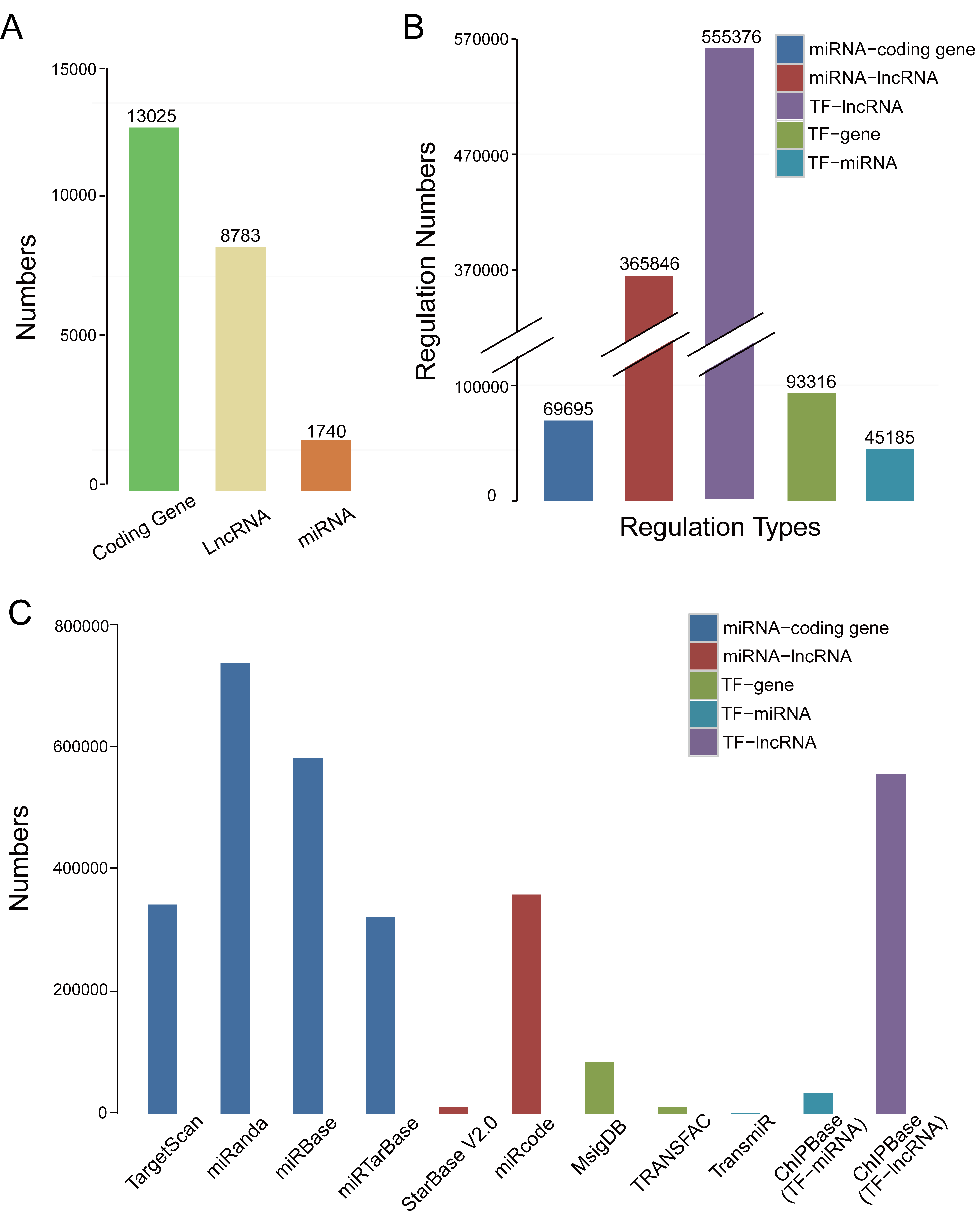
**


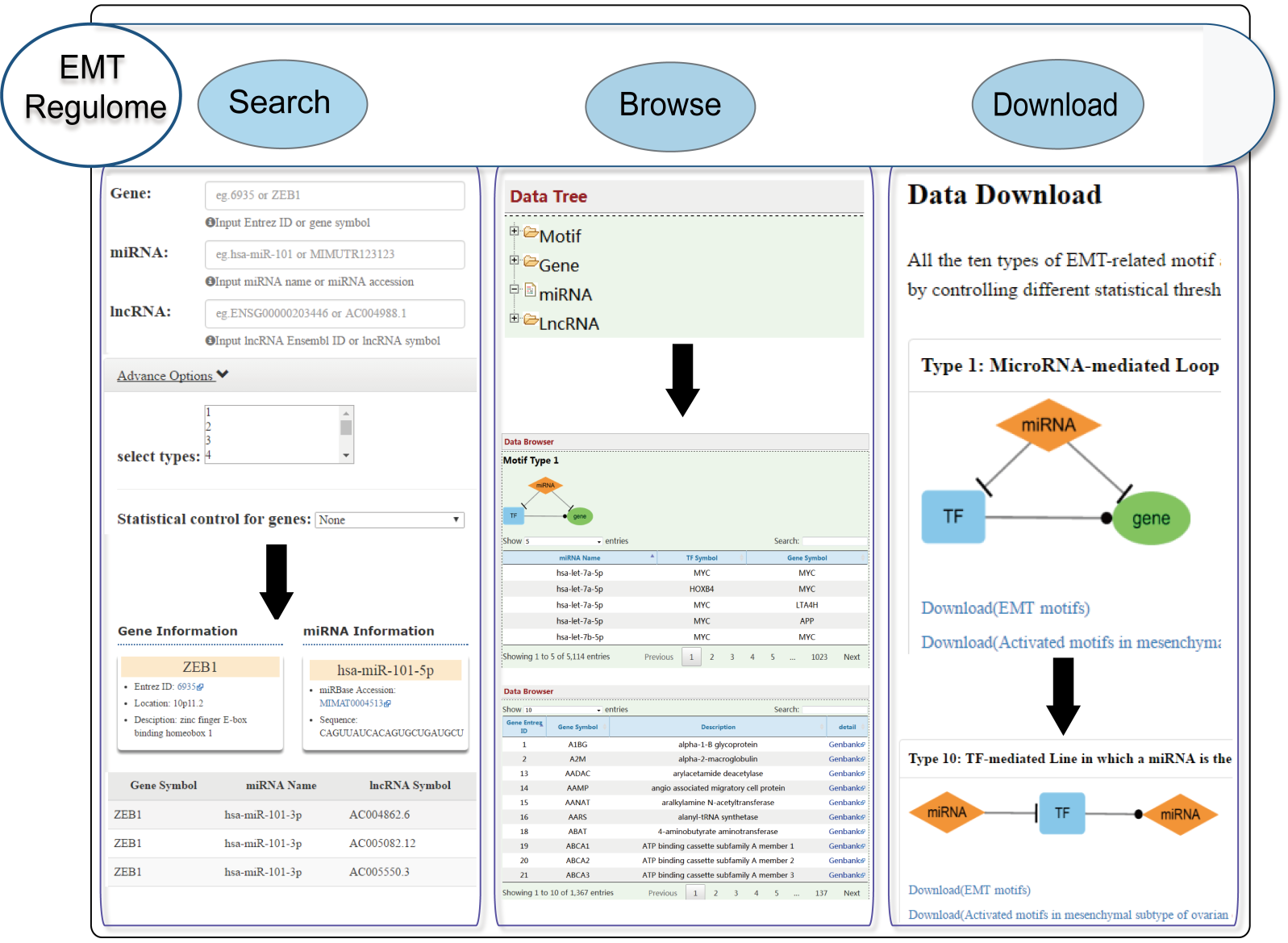
**Supplementary Figure 2 - The overview of EMT-Regulome database.**
